# Brief research report: Repurposing pentoxifylline to treat intense acute swimming–Induced delayed-onset muscle soreness in mice: Targeting peripheral and spinal cord nociceptive mechanisms

**DOI:** 10.3389/fphar.2022.950314

**Published:** 2023-01-10

**Authors:** Sergio M. Borghi, Tiago H. Zaninelli, Telma Saraiva-Santos, Mariana M. Bertozzi, Renato D. R. Cardoso, Thacyana T. Carvalho, Camila R. Ferraz, Doumit Camilios-Neto, Fernando Q. Cunha, Thiago M. Cunha, Felipe A. Pinho-Ribeiro, Rubia Casagrande, Waldiceu A. Verri

**Affiliations:** ^1^ Department of Pathology, Center of Biological Sciences, State University of Londrina, Londrina, Brazil; ^2^ Center for Research in Health Science, University of Northern Paraná, Londrina, Brazil; ^3^ Department of Biochemistry and Biotechnology, Exact Sciences Center, State University of Londrina, Londrina, Brazil; ^4^ Department of Pharmacology, Ribeirão Preto Medical School, University of São Paulo, Ribeirão Preto, Brazil; ^5^ Department of Pharmaceutical Sciences, Center of Health Sciences, State University of Londrina, Londrina, Brazil

**Keywords:** pentoxifylline, muscle mechanical hyperalgesia, cytokine, oxidative stress, glial cells

## Abstract

In this study, we pursue determining the effect of pentoxifylline (Ptx) in delayed-onset muscle soreness (DOMS) triggered by exposing untrained mice to intense acute swimming exercise (120 min), which, to our knowledge, has not been investigated. Ptx treatment (1.5, 4.5, and 13.5 mg/kg; i.p., 30 min before and 12 h after the session) reduced intense acute swimming–induced mechanical hyperalgesia in a dose-dependent manner. The selected dose of Ptx (4.5 mg/kg) inhibited recruitment of neutrophils to the muscle tissue, oxidative stress, and both pro- and anti-inflammatory cytokine production in the soleus muscle and spinal cord. Furthermore, Ptx treatment also reduced spinal cord glial cell activation. In conclusion, Ptx reduces pain by targeting peripheral and spinal cord mechanisms of DOMS.

## Introduction

Delayed-onset muscle soreness (DOMS) is characterized by muscle pain or tenderness occurring upon unaccustomed, eccentric-based, or long-duration exercise (MacIntyre et al., 1995; Cheung et al., 2003; Nicol et al., 2015; Bussulo et al., 2021). Exercise-induced muscle pain is related to the muscle group used in the activity. DOMS is most prevalent at the beginning of a sporting season when athletes return to their activities after a period of inactivity; however, DOMS also has a high prevalence in strenuous and unaccustomed recreational physical activity/exercise (Cheung et al., 2003; Zeng et al., 2022). In a study evaluating students in a prematch training phase of a school sports meeting, the incidence of DOMS reached 72.8% of the total participants (Zeng et al., 2022). Non-steroidal anti-inflammatory drugs are used to treat DOMS (Cheung et al., 2003); however, their efficacy is still controversial (Bourgeois et al., 1999; Connolly et al., 2003; Meamarbashi and Rajabi, 2015; Singla et al., 2015). Other approaches have been recently tested for DOMS treatment, such as analgesics and natural products–derived antioxidants (Bussulo et al., 2021). The topical use of sodium diclofenac 1% (Singla et al., 2015) and, for instance, oral polyphenol curcumin successfully inhibited DOMS (Drobnic et al., 2014; Nicol et al., 2015) induced by dynamic and eccentric exercises. Even though pharmacological approaches have been under investigation for DOMS, its treatment requires novel/alternative pharmacological approaches.

Pentoxifylline (Ptx), a methylxanthine derivative, acts as a phosphodiesterase inhibitor that inactivates intracellular second messengers such as cyclic adenosine monophosphate (cAMP) that modulates gene transcription. Ptx is considered an effective compound in the treatment of immunological diseases where tumor necrosis factor-α (TNF-α) plays a role (Huizinga et al., 1996; Sampaio et al., 1998). Ptx reduces the half-life of TNF-α mRNA, attenuating its production (Strieter et al., 1988; Sampaio et al., 1998; Verri et al., 2006). Ptx also inhibits mRNA and protein levels of interleukin-1β (IL-1β) (Wei et al., 2009) and is an antioxidant (Bhat and Madyastha, 2001). These properties have been attributed to the anti-inflammatory and analgesic effects of Ptx (Dorazil-Dudzik et al., 2004; Vale et al., 2004; Wei et al., 2009; Kim et al., 2016).

Muscle pain observed in DOMS involves the local production of cytokines, such as TNF-α (Borghi et al., 2014b; Dos Santos et al., 2020) and IL-1β (Borghi et al., 2014a) with a hyperalgesic function and IL-10 (Borghi et al., 2015; Alvarez et al., 2017) as an anti-hyperalgesic cytokine. In fact, anti-TNF, IL-1ra, and IL-10 pharmacological approaches reduce DOMS (Borghi et al., 2014a; Borghi et al., 2014b; Borghi et al., 2015). TNF-α and IL-1β contribute to the recruitment of neutrophils that produce reactive oxygen species (ROS), adding to DOMS mechanisms contributing to hyperalgesia (Aoi et al., 2004; Bussulo et al., 2021). DOMS also triggers spinal cord neuroinflammation leading to pain mechanisms, which include TNF-α production and activation of neurons and glial cells (Pereira et al., 2015; Dos Santos et al., 2020; Borghi et al., 2021).

Swimming exercise is a common type of physical activity performed daily, comprising both concentric/eccentric actions. Using long-duration exercise models of DOMS adds to the literature of data from exclusively eccentric exercise DOMS models. Ptx is an FDA-approved drug for the treatment of patients with intermittent claudication related to chronic occlusive arterial disease of the limbs (drug approval package with application no. 74-962 from 1999). Ptx has been marketed for other purposes such as leprosy (Legendre et al., 2012). Ptx has been tested in several preclinical rodent models of neuropathic pain, such as chemotherapy, chronic constriction injury, and spinal nerve transection (Liu et al., 2007; Mika et al., 2007; Kim et al., 2016); inflammatory pain (Vale et al., 2004); radiation-induced pain (Price et al., 2021); and bone fracture–induced complex regional pain syndrome (Wei et al., 2009). Ptx has also shown positive effects in the treatment of human lumbar disc herniation–induced radicular pain (Tarabay et al., 2022). Thus, considering the parallels in the main mechanism of action of Ptx (reduction of TNF-α mRNA half-life) (Strieter et al., 1988; Sampaio et al., 1998; Verri et al., 2006) and DOMS physiopathology with the role of TNF-α (Borghi et al., 2014b; Dos Santos et al., 2020), we reasoned that Ptx would be a potential drug for DOMS treatment. However, to our knowledge, the activity and mechanisms of Ptx have not been previously investigated in DOMS, which is the novel point we aimed to address in this study.

## Materials and methods

**FIGURE 1:**
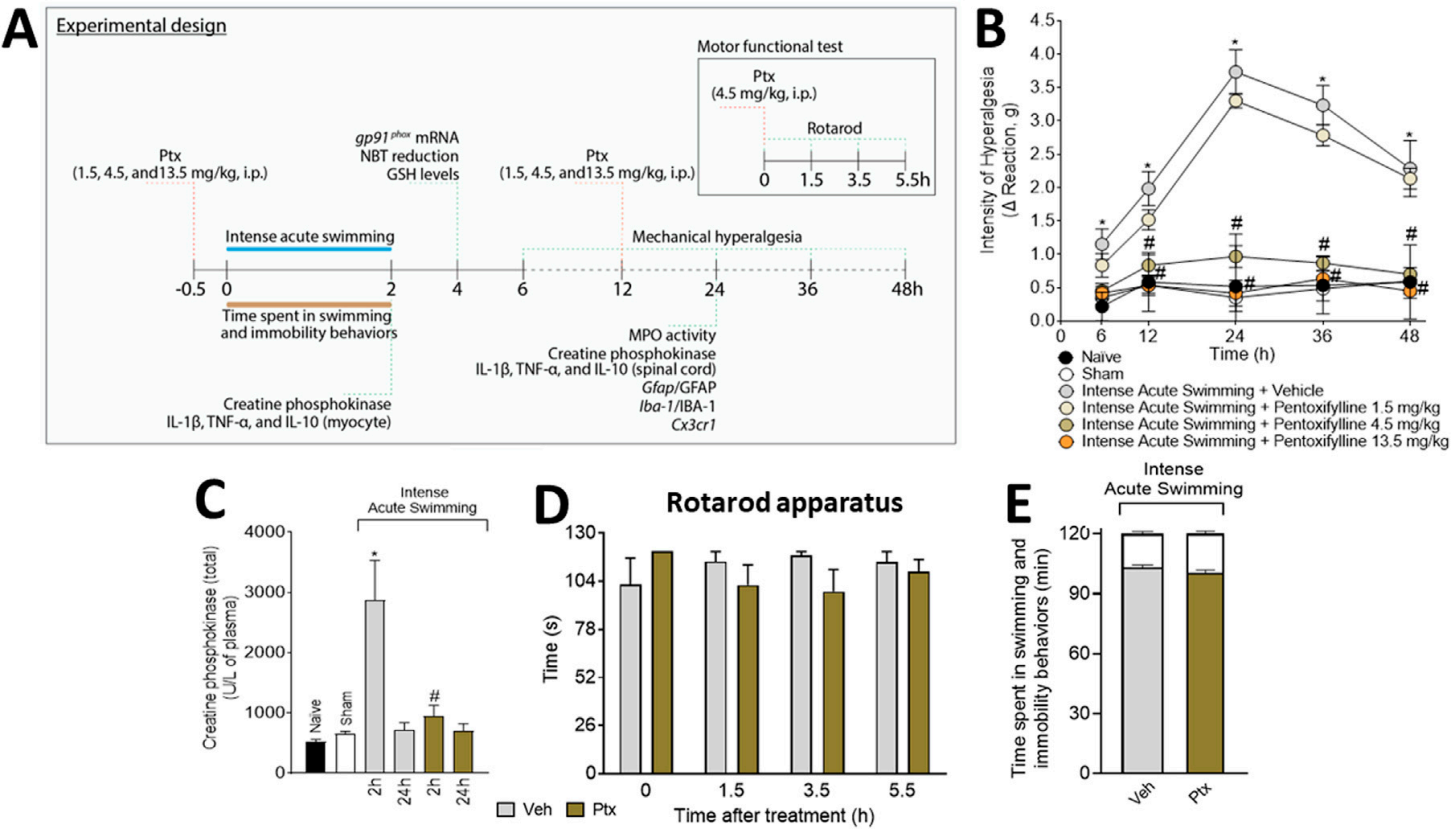
Experimental protocol and analgesic effect of pentoxifylline (Ptx). **(A)** Schematic representation of the experimental protocol used in the study. Ptx inhibits intense acute swimming–induced **(B)** muscle mechanical hyperalgesia and **(C)** increased plasmatic creatine phosphokinase (4.5 mg/kg dose; i.p.), and it does not induce **(D)** motor dysfunctions or impairments in **(E)** time spent in swimming behavior (the white color at the top of the bars indicates the immobility time). Results are presented as intensity of hyperalgesia (Δ reaction, in grams) and as creatine phosphokinase (total) (U/L of plasma) for CK plasmatic levels, and the results of motor and swimming performance are presented in seconds and minutes, respectively (*n* = 6 mice per group per experiment and is representative of two independent experiments). *p < .05 when compared with control groups, #*p* < .05 when compared with the Veh group (two-way ANOVA followed by Tukey’s *post hoc*).

For detailed materials and methods, please refer to the Supplemental Material. Briefly, Figure 1A shows the experimental protocols in which mice were exposed to intense acute swimming for 120 min to induce DOMS. The study used 456 pathogen-free C57BL/6 or LysM-eGFP^+^ Tg male mice (7–8 weeks, 20–25 g). The experiments were conducted in six mice per group, except for histochemical techniques (four mice per group). The mice were identified and subsequently randomized. Experimenters and analyzers were always blinded to the treatments. The primary outcome measure was to evaluate the effects of Ptx on DOMS experimental protocol. The mice received Ptx (1.5, 4.5, and 13.5 mg/kg for a dose–response experiment aiming to screen for a better dose for the study) or vehicle (Veh; 0.9% sterile sodium chloride saline) treatments by the i.p. route at two time points, considering the duration of the experiments. The definition of the Ptx dose range to be tested was based on a previous study by our group (Carvalho et al., 2015), and the selection of the Ptx dose for the mechanistic studies was based on the dose-dependent results of the present study. Mechanical hyperalgesia was assessed using an electronic esthesiometer (Borghi et al., 2014a). The motor function was evaluated using a rotarod (Rustay et al., 2003). Tissue samples were from the soleus muscle or spinal cord (L_4_–L_6_) because DOMS alterations occur in these tissues (Borghi et al., 2014a; Borghi et al., 2014b; Borghi et al., 2015; Borghi et al., 2016; Borghi et al., 2021). Colorimetric assays were used for CK, superoxide anion, GSH, and MPO activity quantitation (Borghi et al., 2014b; Borghi et al., 2016; Borghi et al., 2021). Fluorescence analysis for neutrophil migration was performed using LysM-eGFP^+^ mice (Ruiz-Miyazawa et al., 2018). Cytokines were quantitated by ELISA (Borghi et al., 2015; Borghi et al., 2021). Glial markers were quantitated by RT-qPCR (Borghi et al., 2016) and immunofluorescence (Borghi et al., 2021). The two-way ANOVA was used to compare the groups and doses at all time points. Upon significant time *versus* treatment interaction, the one-way ANOVA followed by Tukey’s *t*-test was used. The results are presented as mean ± standard error of the mean (SEM) for assessments performed on 4–6 mice per group in each experiment depending on the methods and are representative of two separate experiments. The statistical differences are significant at *p* < .05.

## Results

### Ptx treatment inhibits muscle mechanical hyperalgesia and plasmatic CK levels induced by intense acute swimming without impairment of mice motor function or swimming behavior

The i.p. treatment with 1.5 mg/kg of Ptx did not alter muscle mechanical hyperalgesia, but doses of 4.5 and 13.5 mg/kg of Ptx abolished muscle mechanical hyperalgesia (Figure 1B). The dose of 4.5 mg/kg was selected for the next sets of experiments because it achieved a maximal response. Ptx treatment inhibited the plasmatic increase of CK at 2 h (Figure 1C), at a previously selected time point for DOMS (Borghi et al., 2016). Ptx did not alter motor function in the mice (Figure 1D) nor the time spent in swimming/immobility behaviors (Figure 1E).

### Ptx treatment inhibits neutrophil recruitment, cytokine production, and oxidative stress in soleus muscle

**FIGURE 2:**
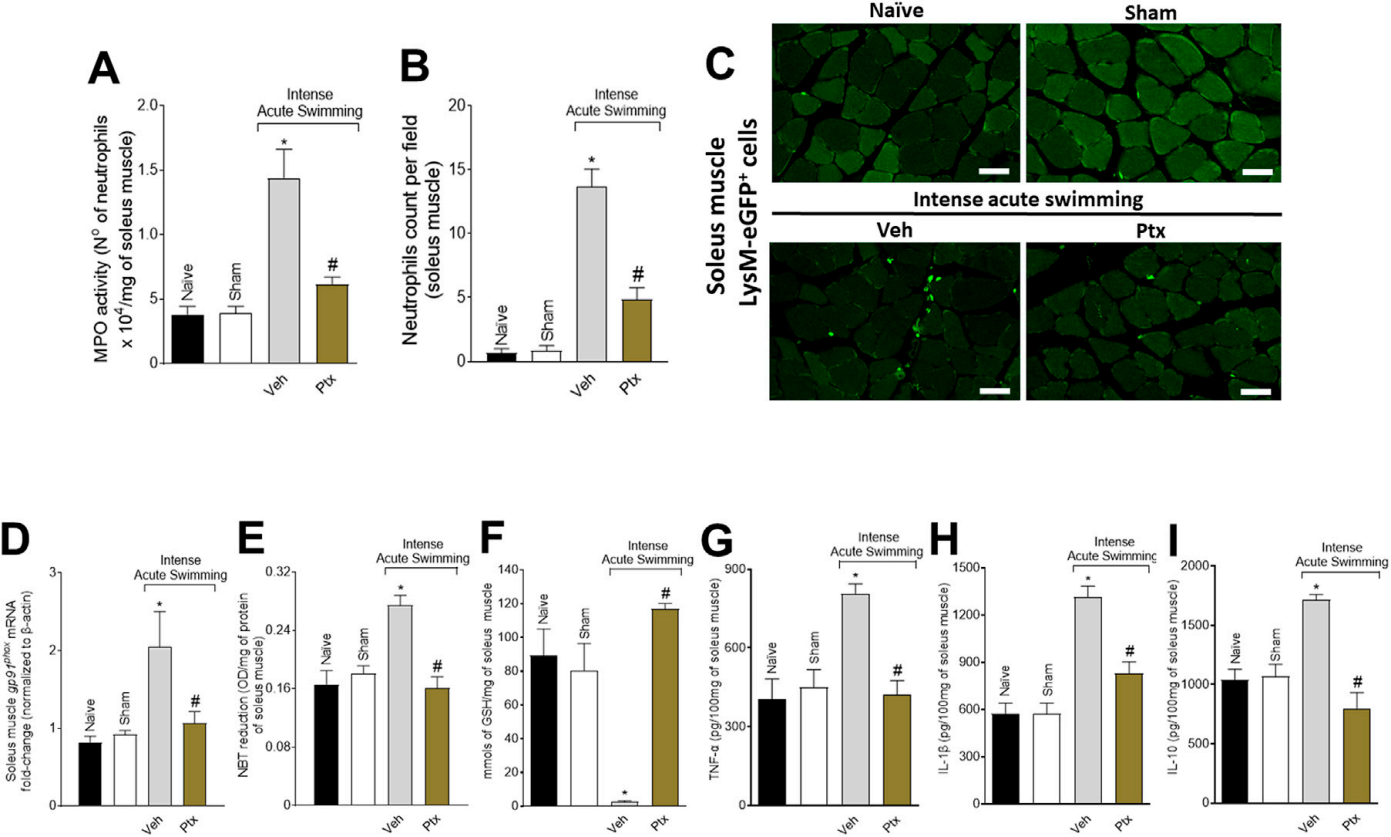
Ptx reduces DOMS recruitment of neutrophils, oxidative stress, and cytokine production in the soleus muscle. Ptx (4.5 mg/kg dose; i.p.) inhibits intense acute swimming–induced **(A)** MPO activity, **(B,C)** LysM-eGFP^+^ neutrophil recruitment, **(D)** *gp91* ^*phox*^ mRNA expression, **(E)** superoxide anion production, **(F)** GSH, **(G)** TNF-α, **(H)** IL-1β, and **(I)** IL-10 levels. Results are presented as the number of neutrophils ×10^4^ per milligram of muscle for MPO activity, neutrophil counts per field, muscle mRNA fold-change normalized to *β-actin* for *gp91* ^*phox*^ expression, as NBT reduction (OD per milligram of protein of muscle for superoxide anion production), millimols per milligram of muscle for GSH levels, and picograms per 100 mg of muscle for cytokine levels (*n* = 6 mice per group per experiment and is representative of two independent experiments). Scale bar: 50 µm *p < .05 when compared with control groups, #*p* < .05 when compared with Veh group (one-way ANOVA followed by Tukey’s *post hoc*).

The MPO activity (Figure 2A) and LysM-eGFP^+^ fluorescent mouse model (Figures 2B, C) revealed that the DOMS + Veh group presented a higher number of neutrophils than the control groups, which was amenable by Ptx treatment. Ptx treatment also reverted the pro-oxidative tissue environment in DOMS characterized by an increase in *gp91* ^*phox*^ mRNA expression (NADPH oxidase subunit expressed by neutrophils) (Borghi et al., 2016) (Figure 2D), superoxide anion production (NBT assay, a NADPH oxidase product) (Figure 2E), and a reduction in GSH levels (an endogenous antioxidant) (Figure 2F). The recruitment of neutrophils and oxidative stress in DOMS were dependent on cytokine production. We observed that Ptx reduced the production of TNF-α (Figure 2G), IL-1β (Figure 2H), and IL-10 (Figure 2I) in DOMS.

### Ptx inhibits cytokine production and glial activation in spinal cord

**FIGURE 3:**
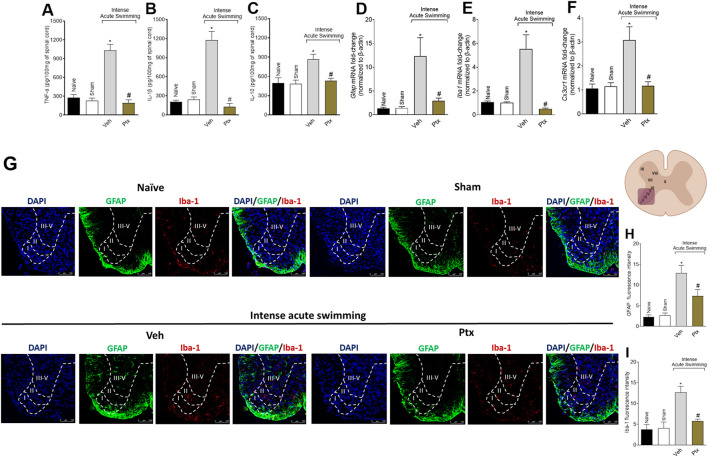
Ptx reduces cytokine production and glial cell activation in the spinal cord. Ptx (4.5 mg/kg dose; i.p.) inhibits intense acute swimming–induced **(A)** TNF-α, **(B)** IL-1β, and **(C)** IL-10 protein levels; **(D)** Gfap, **(E)** Iba1, and **(F)** Cx3cr1 mRNA expression; and GFAP **(G,H)** and **(G,I)** Iba-1 immunofluorescence intensity in the spinal cord. Boundaries of dorsal horn laminae (I–V) were delineated to better clarify the regions of staining. An illustration of the spinal cord in the right denotes the areas in which staining was quantified during the experiments. Results are presented as picograms per 100 mg of the spinal cord for cytokines, as the spinal cord mRNA fold-change normalized to *β-actin* for glial markers mRNA expression (*n* = 6 mice per group per experiment and is representative of two independent experiments), and as fluorescence intensity for glial protein expression (*n* = 4 mice per group per experiment and is representative of two independent experiments; scale bars: 100 µm for the inserts). *p < .05 when compared with control groups, #*p* < .05 when compared with Veh group (one-way ANOVA followed by Tukey’s *post hoc*).

Ptx treatment inhibited the intense acute swimming–triggered production of TNF-α (Figure 3A), IL-1β (Figure 3B), and IL-10 (Figure 3C) in the spinal cord. Ptx also reduced the mRNA expression of glial markers Gfap (Figure 3D), Iba1 (Figure 3E), and Cx3cr1 (Figure 3F), and the immunofluorescence intensity of GFAP (Figures 3G, H) and Iba-1 (Figures 3G, I) in the spinal cord dorsal horn, especially in lamina I and lamina II, where peptidergic C and Aγ myelinated nociceptors synapse with second-order neurons. Lamina I receives input coming from the skeletal muscle (Basbaum et al., 2009; Muir, 2009). Thus, Ptx treatment reduces intense acute swimming neuroinflammation in the spinal cord.

## Discussion

We sought to investigate the analgesic effect and mechanisms of action of Ptx in a DOMS mouse model triggered by intense acute swimming. In the soleus muscle, DOMS induced the production of hyperalgesic cytokines and ROS and recruitment of neutrophils. In the spinal cord, DOMS caused neuroinflammation, which was observed as enhanced cytokine production and glial cells activation. Ptx inhibited all these parameters, which explains its analgesic activity. The efficacy of Ptx was observed when using the dose of 4.5 mg/kg, which is a safe dose since its lethal dose 50 (LD50) is 195 mg/kg/i.p. in mice (Pedretti et al., 2020), and 50 mg/kg/i.p. of Ptx does not induce liver or kidney damage in mice (Inacio et al., 2020).

An increase of CK levels in the blood is a characteristic of DOMS and reflects muscle damage since it is an indicator to the release of creatine kinase into the circulation after disruptions to muscle sarcolemma integrity (Hotfiel et al., 2018). The mechanisms related to CK clearance from the blood are not fully understood, but presumably reflect the complex interactions related to the energy status and magnitude of muscle damage. Its measurements denote the relative amounts of CK released, the degree of this released enzyme activity, and the rate of CK clearance from the serum (Baird et al., 2012). Muscle damage induces inflammation. The MPO enzyme can be found in both neutrophils and macrophages, and the leukocytes that play important roles in the pathophysiology of DOMS (MacIntyre et al., 1995; Bussulo et al., 2021). Ptx reduced CK levels, representing diminished muscle damage, aligning it with reduced muscle inflammation (MPO activity and LysM^+^ cell recruitment). Interestingly, Ptx improves blood flow through the blood vessels (Vale et al., 2004; Verri et al., 2006), an activity that may enhance CK clearance. Importantly, Ptx did not alter the motor performance, time spent swimming, nor immobility time, thus, corroborating that changes in mice response were due to Ptx analgesic activity. Nevertheless, it is important to mention that evidence also supports that inflammation in response to muscle damage is sufficient but not essential for inducing DOMS (Murase et al., 2010; Murase et al., 2013; Shimodaira et al., 2019). These data were obtained from prior models of exclusively eccentric exercise of the gastrocnemius muscle and are, therefore, of conditions that are different from those of the present model (Borghi et al., 2014a; Borghi et al., 2014b; Borghi et al., 2015), thus suggesting that every DOMS model might represent specific conditions, bringing different contributions to understand DOMS physiopathology and pharmacology.

Models of eccentric exercise are classically used for DOMS since the stretching associated with the contraction of the myocytes damages the fibers, and the present model does not exclusively present this feature. We previously developed the intense acute swimming–induced DOMS model (Borghi et al., 2014a; Borghi et al., 2014b; Borghi et al., 2015; Borghi et al., 2016; Borghi et al., 2021) as it relies on a common type of physical activity performed daily, comprising both concentric and eccentric action. Besides affecting athletes, DOMS may affect sedentary people and amateur athletes. DOMS may have a potentially negative impact on the postexercise recovery period and can impair adherence and permanence in physical activity programs, which are harmful within the context of health promotion (Lima et al., 2017). Thus, using long-duration exercise models of DOMS adds to the literature data from exclusively eccentric exercise models of DOMS.

Intense acute swimming induces alterations in the soleus muscle but not in the gastrocnemius (Borghi et al., 2014a; Borghi et al., 2014b; Borghi et al., 2015; Borghi et al., 2016), thus we focused on the soleus muscle. As we observed the recruitment of LysM^+^ neutrophils, we assessed the muscle levels of cytokines and ROS, which are inflammatory mediators of DOMS involved in neutrophil recruitment (MacIntyre et al., 1995; Borghi et al., 2015; Bussulo et al., 2021). Strategies targeting cytokines and ROS have demonstrated that TNF-α, IL-1β, and ROS are hyperalgesic mediators in DOMS (Borghi et al., 2014a; Borghi et al., 2014b; Borghi et al., 2016). IL-10 works as a limiting factor controlling both pro-hyperalgesic cytokine and ROS in DOMS (Borghi et al., 2015). IL-10 is co-released with pro-hyperalgesic molecules to limit their activity. When there is a reduction of inflammation, IL-10 production may also be diminished (Verri et al., 2006; Borghi et al., 2015). The present data suggest that Ptx activity does not depend on increasing IL-10 levels. This triad of phagocytes, cytokines, and ROS is hyperalgesic and interacts with each other (Ming et al., 1987; Jimenez-Altayo et al., 2006; Cunha et al., 2008; Kilpatrick et al., 2010). In the beginning, cytokines and ROS are important to chemoattract neutrophils, but upon arriving at the inflammatory foci, they can produce additional cytokines and ROS thereby enhancing inflammation and pain (Verri et al., 2006; Cunha et al., 2008; Borghi et al., 2014b).

Soleus muscle inflammation activates the primary afferent neurons to induce pain in DOMS (Borghi et al., 2021). It is understood that DOMS pathophysiology starts with ultrastructural damages of the muscle fibers, triggering subsequent inflammation, resulting in the production of inflammatory mediators by resident and recruited immune cells. Inflammatory mediators produced by leukocytes, which include prostaglandins, cytokines, and ROS, lead to the sensitization and activation of group III and IV muscle nociceptor sensory neurons, leading to the sensation of DOMS (Armstrong, 1984; MacIntyre et al., 1995; Cheung et al., 2003; Bussulo et al., 2021). Upon binding of inflammatory mediators to their receptors, activation of the receptors is triggered, leading to neuronal intracellular signaling cascades that vary depending on the inflammatory mediator and type of receptor. These intracellular signaling pathways include protein kinases such as protein kinase A (PKA), protein kinase C (PKC), and mitogen-activated protein kinases (MAPK) that sensitize ion channels, enhancing their opening frequency and time in open conformation. This raises the ion concentration (e.g., Na^+^) inside neurons, causing their depolarization/activation or enhancing responses to other stimuli such as mechanical stimulation. For instance, TNF-α acting on TNFR1 can induce peripheral mechanical sensitization in primary afferent neurons through p38 MAPK-dependent modulation of tetrodotoxin-resistant (TTX-R) Na^+^ channels (Jin and Gereau, 2006). Increased TTX-R currents (enhanced by prostaglandin E2, for example) potentiate sodium entry into the nociceptor sensory neurons, boosting action potentials (Blair and Bean, 2002). Altogether, these events account for DOMS peripheral sensitization. After receiving peripheral inputs, the central branches of these muscle nociceptor sensory neurons likely release chemokines such as CX_3_CL1 in the spinal cord that activate the microglia. This mechanism interlinks the peripheral and central nervous systems in the spinal cord causing glial activation, and glial–glial and glial–neuron interactions, with consequent spinal cord neuroinflammation (Souza et al., 2013; Pinho-Ribeiro et al., 2017). We have previously demonstrated that intense acute swimming leads to increased Cx3cl1 mRNA expression in the spinal cord of mice with DOMS, and there being an increase of CX_3_CR1, treatment with an anti-CX_3_CL1 antibody reduces DOMS (Borghi et al., 2021). CX_3_CL1 release from the neurons depends on prior activation of the glial cells in the spinal cord that can be triggered by the neuronal release of adenosine triphosphate (ATP). Once activated, the glial cells can induce CX_3_CL1 release by the neurons *via* ADAM10, ADAM17, or cathepsin S activity (Clark and Malcangio, 2012; Silva and Malcangio, 2021). These conclusions have been drawn from the data on varied models; however, the role of proteases in CX_3_CL1 release in DOMS spinal cord neuroinflammation remains to be determined. Targeting cytokines, chemokines, and glial cells in the spinal cord is effective in reducing intense acute swimming DOMS (Borghi et al., 2016; Borghi et al., 2021), and Ptx treatment reduces spinal cord neuroinflammation.

Evidence supports that Ptx acts by reducing the half-life of TNF-α mRNA, which results in reduced TNF-α production. A part of Ptx activity is at the transcriptional level (Doherty et al., 1991; Han et al., 1991). Ptx is a methylxanthine, and its activity is shared by some methylxanthine and dibutyryl cAMP (Strieter et al., 1988). Ptx inhibits phosphodiesterase activity, thereby increasing intracellular cAMP, which reduces TNF-α release (Verghese et al., 1995). However, this activity is not exclusive for TNF-α since other cytokines can be targeted by Ptx (Verri et al., 2006). Thus, it is a multitarget drug that can interfere with the processes of producing and releasing cytokines, TNF-α included. By inhibiting TNF-α production, Ptx can reduce inflammation, nociceptor neuron activation and sensitization that lead to DOMS, and neurons–glial cells communication in the spinal cord that depends on nociceptor neuronal activation. Peripheral administration of Ptx can induce a therapeutic effect in the brain (Eun et al., 2000). In this sense, the activity of Ptx in the spinal cord to reduce neuroinflammation could also be direct in this site (Mika et al., 2007; Mika et al., 2009; Saito et al., 2010; Bas et al., 2012) and possibly with the involvement of Ptx metabolites that are also pharmacologically active (Fantin et al., 2006). Thus, Ptx targets varied levels of the nociceptive process.

In summary, Ptx has been shown to inhibit TNF-α and IL‐1β synthesis (Strieter et al., 1988; Neuner et al., 1994; Sampaio et al., 1998; Verri et al., 2006; Wei et al., 2009) and reduce oxidative stress (Bhat and Madyastha, 2001; Vircheva et al., 2010). Moreover, Ptx inhibits microglia activation (Chao et al., 1992; Mika et al., 2007), which might account for counteracting spinal cord inflammation and sensitization, by inhibiting glial–glial and neuron–glia interactions (Pinho-Ribeiro et al., 2017). Thus, since TNF-α and IL-1β, and oxidative stress are crucial aspects for peripheral sensitization, while glial activation is important to central sensitization, we hypothesize that by both peripheral and central mechanistic inhibitory effects, Ptx inhibits DOMS pathophysiology.

**FIGURE 4:**
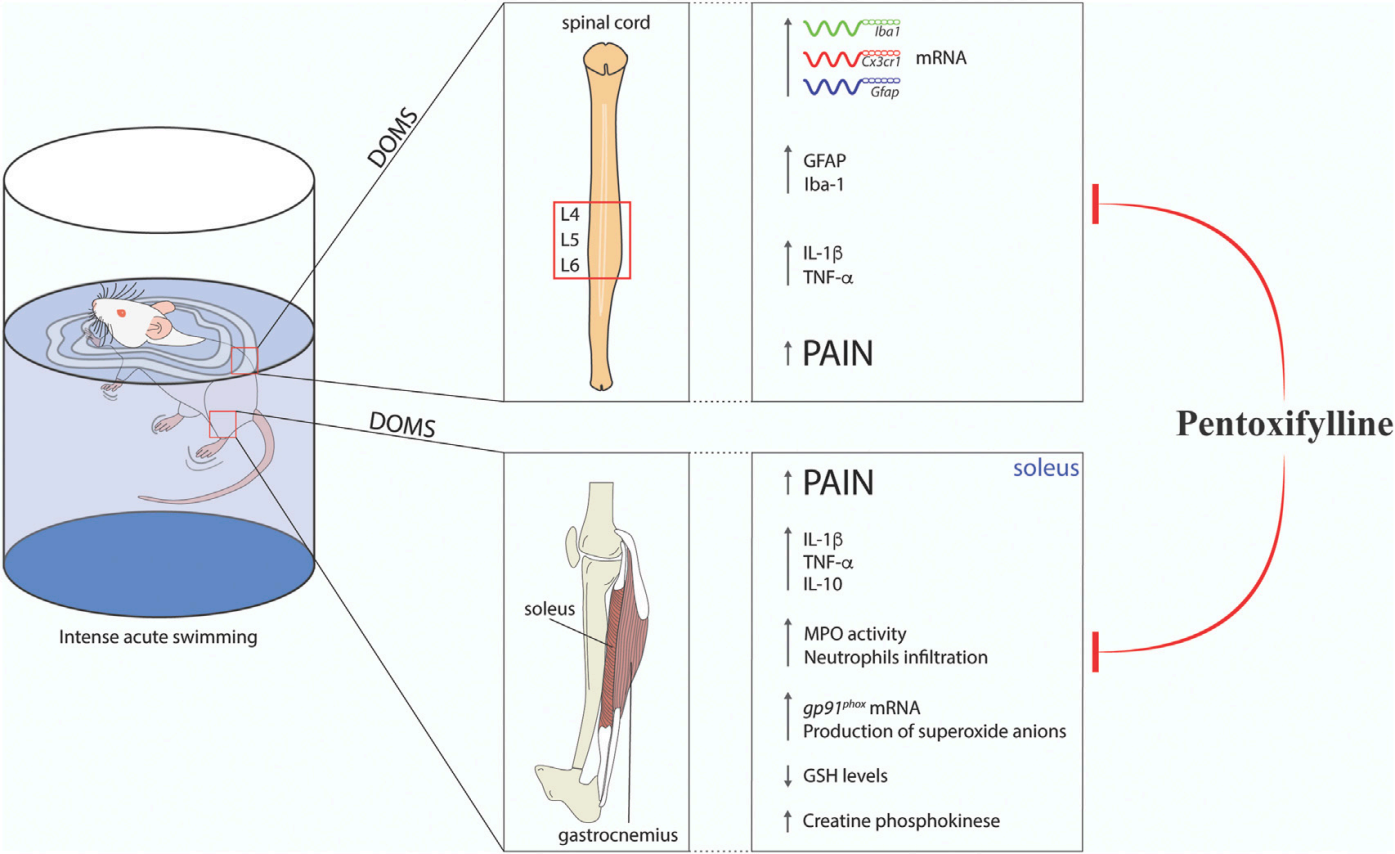
Schematic representation of pentoxifylline modulatory mechanisms in experimental DOMS in mice. Ptx treatment reduced DOMS CK elevation, neutrophils recruitment, oxidative stress (increased *gp91* ^*phox*^ mRNA expression and superoxide anion production and reductions in GSH levels), and cytokine production (TNF-α, IL-1β, and IL-10 levels) in the soleus muscle. In the spinal cord, Ptx treatment inhibited cytokine production (TNF-α, IL-1β, and IL-10 levels) and astrocytes and microglial activation at the mRNA (Gfap, Iba1, and Cx3cr1) and protein (GFAP and Iba-1) levels. These integrated peripheral and spinal mechanisms lead to the reduction of muscle pain (DOMS) by Ptx treatment.

Clinically, Ptx is used to treat muscle pain in patients with peripheral arterial diseases (Broderick et al., 2020) and reduces radiculopathy-related pain (Tarabay et al., 2022). In terms of translation to humans, these preclinical data add to the literature the potential applicability of Ptx for the treatment of musculoskeletal pain, such as DOMS. Figure 4 shows the findings of the present study. To our knowledge, this is the first evidence that Ptx can be repurposed for the treatment of DOMS since it reduces peripheral muscle inflammation and spinal cord neuroinflammation.

## Data Availability

The raw data supporting the conclusions of this article will be made available by the authors, without undue reservation.
