# Supplementary material for: Brief research report: Repurposing pentoxifylline to treat intense acute swimming–Induced delayed-onset muscle soreness in mice: Targeting peripheral and spinal cord nociceptive mechanisms
Source: Front Pharmacol. 2023 Jan 10;13:950314. doi: 10.3389/fphar.2022.950314 (PMC9871252; doi:10.3389/fphar.2022.950314)
Supplement: Supplementary file 1 [file Presentation1.pdf]

Supplementary Data 1

Repurposing pentoxifylline to treat intense acute swimming-induced delayed-onset muscle soreness in mice: Targeting peripheral and spinal cord nociceptive mechanisms

Sergio M. Borghi,[1,2,*] Tiago H. Zaninelli,[1] Telma Saraiva-Santos,[1] Mariana M. Bertozzi,[1] Renato D. R. Cardoso,[1] Thacyana T. Carvalho,[1] Camila R. Ferraz,[1] Doumit Camilios-Neto,[3] Fernando Q. Cunha,[4] Thiago M. Cunha,[4] Felipe A. Pinho-Ribeiro,[1] Rubia Casagrande,[5] Waldiceu A. Verri Jr[1,*]

[1]Department of Pathology, Center of Biological Sciences, State University of Londrina, Londrina, Brazil.

[2]Center for Research in Health Science, University of Northern Paraná, Londrina, Brazil.

[3]Department of Biochemistry and Biotechnology, Exact Sciences Center, State University of Londrina, Londrina, Brazil.

[4]Department of Pharmacology, Ribeirão Preto Medical School, University of São Paulo, Ribeirão Preto, Brazil.

[5]Department of Pharmaceutical Sciences, Center of Health Sciences, State University of Londrina, Londrina, Brazil.

[*]Authors to whom correspondence should be addressed: Prof. Sergio M. Borghi, Centro de Pesquisa em Ciências da Saúde, Universidade Norte do Paraná, Rua Marselha, 591, Jardim Piza, 86.041-140, Londrina, Paraná, Brasil; Tel: + 55 43 3371-7990; Mobile: + 55 43 99918-9216; E-mail address: sergio_borghi@yahoo.com.br or sergio.borghi@kroton.com.br; and Prof. Waldiceu A. Verri Jr, Centro de Ciências Biológicas Departamento de Ciências Patológicas, Rod. Celso Garcia Cid, Pr 445, Km 380, Postal Code 10.011, 86057-970, Londrina, Paraná, Brasil; Tel. + 55 43 3371-4979; E-mail address: waldiceujr@yahoo.com.br or waverri@uel.br

Running title: Pentoxifylline reduces DOMS in mice.

Supplementary Data 1

Animals

The experiments were performed on pathogen-free male C57BL/6 mice, 7-8 weeks, 21-25g from State University of Londrina (UEL), Londrina, Paraná State, Brazil, and LysM-eGFP$^+$ mice (20-25 g, C57BL/6 background) from Ribeirão Preto Medical School, University of São Paulo, Ribeirão Preto, São Paulo, Brazil. Mice were housed in standard clear plastic cages with free access to water and food, light/dark cycle of 12/12h, and controlled temperature (22 ±1 °C), with air exhaustion and humidity between 50-60%. The mice were reared in the central vivarium of UEL and kept in the vivarium of the Department of Pathology of the same University for at least two days before experiments. Mice (*n* = 4-6 per group) were used only once and were acclimatized to the behavior testing room at least 1 hour before the experiments, which was conducted always during the light cycle. Animals' care and handling procedures were in accordance with the International Association for Study of Pain (IASP) guidelines and with the approval of the Ethics Committee in the Use of Animals (CEUA), process number 2066.2011. For sample collection, mice were anesthetized with isoflurane 5% only once by inhalation overdose, followed by cervical dislocation and decapitation procedures. A total of 456 animals were used during the study. All efforts were made to minimize the number of animals used and their suffering.

Chemicals, treatment routes and experimental design

Drugs were obtained from the following sources: Ptx (Trental®) was purchased from Hoecht do Brasil Química Farmacêutica (São Paulo, São Paulo, Brazil) and saline solution 0,9% (vehicle; Veh) from Gaspar Viana S/A (Fortaleza, Ceará, Brazil). Ptx was

dissolved in DMSO (20%) for intra-peritoneal (i.p.) and intrathecal (i.t.) treatments and isotonic saline solution (NaCl 0,9%) (80%) immediately before use. Ptx i.p. treatment was conducted in two time periods, 30 min before and 12 h after the intense acute swimming session. This treatment protocol was based on the treatments used to inhibit TNF-α in DOMS (Borghi et al., 2014b). As the aim of this study was to demonstrate the proof-of-concept whether Ptx could potentially be repurposed for DOMS treatment, we first focused on verifying its activity using a known protocol that targets TNF-α in DOMS. I.t. injections were conducted in unconscious mice (targeting L_4-L_6 segment) under anesthesia with isoflurane (3% inhalation). I.t. treatment was performed only once to avoid local inflammation and enhancement of nociception (Almeida et al., 2000). Animals were randomly divided in treatment groups and samples/analyzes were performed at indicated time points described in Fig. 1A. All parameters were previously standardized (Borghi et al., 2014a;Borghi et al., 2014b;Borghi et al., 2016;Borghi et al., 2021). All parameters mentioned were evaluated upon i.p. treatment with Ptx. The Ptx i.t. treatment was used only to demonstrate its spinal cord analgesic effect.

Intense acute swimming protocol to induce DOMS

Mice were placed in a glass box (45×28×25 cm, divided in six compartments) with approximately 20 liters of water at 31° ± 1°C as described previously (Borghi et al., 2014b). Briefly, each mouse was individually placed in one compartment and (Borghi et al., 2014b) swam all the same time during 120 min. After the intense acute swimming session or sham conditions, animals were dried and placed in cages together with their randomized respective group (Borghi et al., 2014a;Borghi et al., 2014b;Borghi et al., 2016;Borghi et al., 2021). This is not a stress protocol as we have already demonstrated (Borghi et al., 2014b).

Evaluation of muscle mechanical hyperalgesia

In a quiet room, mice were placed in acrylic cages (12×10×17 cm) with wire grid floors, 15-30 min before the start of the test. Evaluations consisted of evoking a hind paw flexion reflex with a hand-held force transducer (electronic von Frey anesthesiometer; Insight, Ribeirão Preto, São Paulo, Brazil) adapted with a 0.5 mm^2 contact area polypropylene tip. We used the regular probe of electronic von Frey apparatus, since in a previous publication of our group (Borghi et al., 2014b) we demonstrated that using regular probe (0.5 mm^2 contact area) that elicits nociceptive responses per se, and large probe (4.15 mm^2 contact area) that does not elicit nociceptive responses per se, we obtained equivalent results in intense acute swimming-induced mechanical hyperalgesia, highlighting that the reason by our success in measuring muscle pain with a 0.5mm^2 diameter probe was due to intact cutaneous paw tissue added to simultaneous sensitized skeletal muscle tissue, the latter provoked by muscle overload induced by intense acute swimming DOMS model. During the investigations performed in the experiments, movement-elicited hyperalgesia was provoked by the pressure exerted by the von Frey probe on the plantar surface, which induces the dorsal flexion of the ankle joint (an antagonistic movement provided by the contraction of the soleus muscle), which leads to the passive stretch of the Achilles tendon, generating muscle distention. Thus, the end point of evaluations was always characterized by the removal of the paw followed by clear flinching movements when the muscle of the mice is distended (Borghi et al., 2014a;Borghi et al., 2014b). The measurements were performed only when the animals were not agitated, and with all four paws resting on the floor of the grid. Evaluations occurred between 6 to 48 h after the intense acute swimming session. We established the measurement timeline up to 48 hours in our acute swimming model, as we identified that

the peak of pain-like behavior in the mouse in the present model occurs before that observed in humans (48-72 h), at the 24th hour after the swimming exercise. During the pilot experiments that were conducted at the beginning of the project, significant pain was no longer observed after the 48th hour. For this reason, we determined the cut-off of mechanical hyperalgesia evaluations at 48 h for mice. Eventually, exposition to a longer swimming period could achieve a model that lines up exactly with the human time course of DOMS. Two h of swimming is a considerable exercise duration. The Londrina State University Committee on Animal Welfare understood that this would be reasonable considering species differences, however, longer time periods of exercise would not be justifiable since a similar profile was found. Three repetitions of each measurement were used during the experiment to obtain the average of the final value. The results are expressed by delta (Δ) withdrawal threshold (in g) calculated by subtracting the mean measurements (indicated time points) after stimulus from the baseline measurements. Behavioral analyses were carried out always by the same person, blinded to the treatments.

Evaluation of CK blood concentration

Blood samples were collected after the swimming session, and subsequently centrifuged (1.500 g, 4°C, 10 min), and the resultant plasma was assayed for CK levels according to the manufacturer’s guideline (Ref: 117, Labtest Diagnóstico S.A., Lagoa Santa, MG, Brazil). The results were presented as creatine phosphokinase (U/L of plasma).

Rotarod performance test

The test is used to quantitate the effects of varied conditions and procedures upon motor-planning, with very high reliability (Rustay et al., 2003). The equipment is composed by

a bar (2.5 cm diameter) separated into 4 compartments by disks of 25 cm in diameter (Ugo Basile, model 7600). The evaluations occurs when the bars rotate at a constant speed of 22 rotations (min). Mice were selected 24 h before the treatments by eliminating those that did not remain on the bar for two consecutive periods of 180 seconds. Selected mice were then evaluated at baseline, (0), 1.5, 3.5, and 5,5 h after the treatments with Veh or Ptx. The cutoff time used for al mice in the apparatus was 180 sec.

Evaluation of neutrophils recruitment to the muscle tissue

The intense acute swimming-induced neutrophil recruitment was evaluated at 24 h after the swimming session using MPO kinetic-colorimetric method and immunofluorescence assays (Borghi et al., 2014b;Ruiz-Miyazawa et al., 2018). MPO activity methods was already described (ref). For fluorescence analysis, the soleus muscle was collected after the LysM-eGFP mice were perfused through the ascending aorta with phosphate buffered saline (PBS) followed by 4% of paraformaldehyde (PFA) twice. Samples were subsequently fixed in PFA 4% for 24 h, and after this period, PFA was replaced by a solution of 30% saccharose and incubation for 3 additional days. After this, muscles were washed with PBS and embedded in optimum cutting temperature (O.C.T.) using Tissue-Tek® reagent (Sakura® Finetek USA, Torrance, CA). Sections of 10 micrometers (µm) were cut in a cryostat (CM1520, Leica Biosystem, Richmond, IL, USA) and processed for immunofluorescence (four samples per mice per slide/4 mice per group). For this experiment four samples (sections) per mouse per slide and 6 animals per group were applied. Four experimental groups were analyzed during the experiments, as follows, naïve, sham, vehicle-, and Ptx-treated groups. The representative images and quantitative analysis were created and performed using a confocal microscope (SP8, Leica Microsystems, Mannheim, Germany). Bright field channel was used simultaneously

during acquisitions. Neutrophils count was quantitated in random fields by an experimenter blind to the treatments. The results were presented as neutrophils count per field (of muscle).

Analyses of oxidative stress parameters and gp91^{phox} mRNA expression by reverse transcription and quantitative polymerase chain reaction (RT-qPCR)

Oxidative stress-related parameters (NBT reduction and GSH levels) were assessed in muscle tissue 4 h after the swimming session (Borghi et al., 2016). In addition to those oxidative stress tests, RT-qPCR assay was performed to determine the mRNA expression of gp91^{phox} as previously described (Borghi et al., 2016). Muscle samples were collected 4 h after the swimming session and homogenization in TRIzol™ Reagent (Thermo Fisher Scientific). Total RNA was isolated following the manufacturer's instructions. The purity of total RNA was measured by spectrophotometry, and the wavelength absorption ratio (260/280) was between 1.8 and 2.0 for all preparations during the experiment. Reverse transcription of total RNA to cDNA and qPCR were conducted by GoTaq® 2-Step RT-qPCR System (Promega) and target primer. qPCR reaction was performed in Step One Plus TM Real-Time PCR System (Applied Biosystems®). The relative gene expression was measured using the comparative $2^{-(\Delta\Delta Cq)}$ method. The expression of β-actin mRNA was used as a control for tissue integrity. The sequence of primers used were: *gp91^{phox}*, sense: 5′-AGCTATGAGGTGGTGATGTTAGTGG-3′, antisense: 5′-CACAATATTTGTACCAGACAGACTTGAG-3′, and *β-actin*, sense: 5′-AGCTGCGTTTTACACCCT TT-3′, antisense: 5′-AAGCCATGCCAATGTTGTCT-3′. Nitroblue tetrazolium (NBT) reduction test was used for determining the production of superoxide anion. Samples were collected in 500 µL of saline solution, and 50 µL of the homogenate was transferred to a sterilized 96-well plate. Next, an addition of 100 µL of

NBT solution (1 mg/mL) and incubation for 1 h at 37°C was conducted. The remining supernatant was then removed from plates, and the precipitated formazan in the wells was solubilized by adding 120 μL of 2M KOH and 140 μL of dimethylsulfoxide (DMSO). The concentration of superoxide anion was evaluated by spectrophotometry via the reduction of the redox dye NBT at 600 nm (Multiskan GO Microplate Spectrophotometer, Thermo Fischer Scientific, Vantaa, Finland). The NBT reduction levels were corrected according to the total protein concentration. The results were presented as NBT reduction [optical density (OD)/mg of protein of muscle (Borghi et al., 2021). GSH muscle content assay was also determined using a spectrophotometric method (Borghi et al., 2014b). Muscle samples were initially homogenized in ethylenediaminetetraacetic acid (EDTA) 0,02M. Homogenates were then treated with 2 ml H_20 Milli Q plus 0.5 ml of trichloroacetic acid (TCA) 50%. After 15 min, the homogenates were centrifuged (1500 *g* for 15 min at 4°C), and 1 ml from generated supernatant was added to a 2 ml of a solution containing Tris 0.4M (pH 8.9) plus 50 ml of 5,5′-dithiobis-(2-nitrobenzoic acid) (DTNB) reagent. After additional 5 min, the measurements were carried out at 412 nm (Multiskan GO Microplate Spectrophotometer, Thermo Fischer Scientific, Vantaa, Finland). The results were presented as mmols of GSH/mg of muscle.

ELISA Tests for cytokine production

Muscle and spinal cord (L_4-L_6) samples were collected immediately after (2 h) and 24 h after the swimming session, respectively, for the evaluation of TNF-α, IL-1β and IL-10 production by enzyme-linked immunosorbent assay (ELISA) using eBioscience kits (Affymetrix, San Diego, CA, USA). The technical procedure for collecting the lumbar segments, is related to the identification of the dorsal root ganglia, and then followed back the root to the spinal level using a stereo microscope. Samples were homogenized in

appropriate buffer containing protease inhibitors and properly centrifuged for supernatant generation. During the assay, following initial blocking of the plates, they were coated overnight at 4°C with an immunoaffinity-purified polyclonal antibodies specific for evaluated cytokines. In the next day, recombinant murine diluted TNF-α, IL-1β and IL-10 standards and the samples were added in wells in duplicate and incubated by an additional period of 2 h at room temperature. Rabbit biotinylated immunoaffinity-purified antibodies anti-TNF-α, anti-IL-1β, anti-IL-10 were then added, followed by another incubation at room temperature with duration of 1 h. Subsequently, avidin-HRP was added to wells, and after an additional period of 30 min, the plates were properly washed and the color reagent o-phenylenediamine was added in the concentration of 200 μg per well. After these steps, reactions were blocked and measurements conducted spectrophotometrically at 450 nm (Multiskan GO Microplate Spectrophotometer, Thermo Fischer Scientific, Vantaa, Finland). The results were expressed as pg of each cytokine/100 mg of tissue (Borghi et al., 2015;Borghi et al., 2021).

Evaluation of spinal cord glial cells activation

The activation of spinal cord astrocytes and microglia was assessed by RT-qPCR and immunofluorescence techniques performed 24 h after the swimming session (Borghi et al., 2016;Borghi et al., 2021). For evaluation of GFAP, Iba-1, and CX_3CR1 mRNA expressions, spinal cord samples were processed in the same way as that presented for $gp91^{phox}$ described above (in section analysis of oxidative stress parameters). The sequence of primers used were: *Gfap*, sense: 5′-GGCGCTCAATGCTGGCTTCA-3′, antisense: 5′-TCTGCCTCCAGCCTCAGGTT-3′, *Iba1*, sense: 5′-ATGGAGTTTGATCTGAATGGAAAT-3′, antisense: 5′-TCAGGGCAGCTCGGAGATAGCTTT-3′, and *Cx3cr1*, sense: 5′-

CACCATTAGTCTGGGCGTCT-3′, antisense: 5′-GATGCGGAAGTAGCAAAAGC-3′. The sequence of *β-actin* has been presented earlier. For immunofluorescence approach, mice were perfused (same procedure described in the section of evaluation of neutrophils recruitment to the muscle tissue), and L_4-L_6 segments of the spinal cord were accurately dissected out. Regarding the technical procedure for collecting the lumbar segments. First, the dorsal root ganglia was identified, and then it was traced back the root to the spinal level using a stereo microscope. The fixing and embedding processes were the same as described above in the section of evaluation of neutrophils recruitment to the muscle tissue. Sections (10 μm) were performed in a cryostat (CM1520, Leica Biosystem, Richmond, IL, USA). For this experiment four samples per mouse per slide and 4 animals per group were applied. Initially, sections were blocked with a buffer solution (250 μL per slide containing PBS plus 0.1% tween 20 plus 5% BSA) for 2 h at room temperature. Later, samples were incubated overnight at -4°C with a solution containing primary antibodies against glial cells. After these steps, a new incubation with secondary antibodies against hosts was performed for 1 h at room temperature. For GFAP (#180063, 1:500 dilution; Invitrogen, Life Technologies, Carlsbad, CA, USA) and Iba-1 (#PA5-27436, 1:500 dilution; Invitrogen, Life Technologies, Carlsbad, CA, USA) primary antibodies, Alexa Fluor 488 (#A-110088, 1:1000, Thermo Fischer Scientific, Waltham, MA, USA) and Alexa Fluor 647 (#A32733, 1:1000, Thermo Fischer Scientific, Waltham, MA, USA) secondary antibodies were used, respectively. The assembly of the slides were conducted using ProLong^{TM} Gold Antifade Mountant with DAPI melting media (#P36931, Thermo Fischer Scientific, Waltham, MA, USA). Analysis using slides with secondary antibodies alone was also performed as controls to ensure that unspecified staining did not occur during the experiment. Immunofluorescence analyzes were conducted in the dorsal horn of the spinal cord in the magnification of 20x as indicated in

the figure legends. The quantification of fluorescent intensity of GFAP and Iba-1 spinal cord staining were conducted using as a parameter the Rexed laminae delimitations, presented in illustrative format in Figure 3, and the confocal microscope software (TSC SP8, Leica Microsystems, Mannheim, Germany).

Statistical Analysis

Results are presented as means ± standard error mean (SEM) of assessments performed on 4 or 6 mice in per group during the experiment, depending on the method and are representative of two separated experiments. Two-way analysis of variance (ANOVA) was used to compare the groups and doses at all time points (curves). The analyzed factors were treatments, time, and time versus treatment interaction. When there was a significant time versus treatment interaction, one-way ANOVA followed by Tukey's t-test was performed for each time. Statistical differences were significant at $P < 0.05$. For detailed information on statistical analysis, please see Tables S1, which includes information about t test (*P* values), and one- or two-way ANOVA (F and *P* values).

Summary of statistical analysis

Table S1. Statistical information from results shown in Figures 1-3.

Statistical test	Post-test	F (DFn, DFd) value	P value
Two-way ANOVA			
Fig. 1B	Tukey	Interaction (Time x Dose):	
		F (16, 125) = 7.395	P<0.0001
		Time: F (4, 125) = 26.07	P<0.0001
		Dose: F (4, 125) = 123.6	P<0.0001
One-way ANOVA			
Fig. 1C	Tukey	F (5, 30) = 9.803	P<0.0001
Paired t test (two-tailed)			
Fig. 1D	-	-	P=0.5857
Fig. 1E	-	-	P=0.1698
One-way ANOVA			
Fig. 2A	Tukey	F (3, 20) = 17.13	P=0.0093
Fig. 2B	Tukey	F (3, 20) = 47.18	P<0.0001
Fig. 2D	Tukey	F (3, 20) = 5.370	P=0.0071
Fig. 2E	Tukey	F (3, 20) = 12.69	P<0.0001
Fig. 2F	Tukey	F (3, 20) = 18.28	P<0.0001
Fig. 2G	Tukey	F (3, 20) = 9.667	P=0.0004
Fig. 2H	Tukey	F (3, 20) = 24.95	P<0.0001
Fig. 2I	Tukey	F (3, 20) = 16.00	P<0.0001
One-way ANOVA			
Fig. 3A	Tukey	F (3, 20) = 40.06	P<0.0001
Fig. 3B	Tukey	F (3, 20) = 42.20	P<0.0001
Fig. 3C	Tukey	F (3, 20) = 7.124	P=0.0019
Fig. 3D	Tukey	F (3, 20) = 7.169	P=0.0019
Fig. 3E	Tukey	F (3, 20) = 15.07	P<0.0001
Fig. 3F	Tukey	F (3, 20) = 9.499	P=0.0004
Fig. 3H	Tukey	F (3, 12) = 15.68	P=0.0002
Fig. 3I	Tukey	F (3, 12) = 11.87	P=0.0002

References

Almeida, F.R., Schivo, I.R., Lorenzetti, B.B., and Ferreira, S.H. (2000). Chronic intrathecal cannulation enhances nociceptive responses in rats. *Braz J Med Biol Res* 33**,** 949-956.

Borghi, S.M., Bussulo, S.K.D., Pinho-Ribeiro, F.A., Fattori, V., Carvalho, T.T., Rasquel-Oliveira, F.S., Zaninelli, T.H., Ferraz, C.R., Casella, A.M.B., Cunha, F.Q., Cunha, T.M., Casagrande, R., and Verri, W.A., Jr. (2021). Intense Acute Swimming Induces Delayed-Onset Muscle Soreness Dependent on Spinal Cord Neuroinflammation. *Front Pharmacol* 12**,** 734091.

Borghi, S.M., Pinho-Ribeiro, F.A., Fattori, V., Bussmann, A.J., Vignoli, J.A., Camilios-Neto, D., Casagrande, R., and Verri, W.A., Jr. (2016). Quercetin Inhibits Peripheral and Spinal Cord Nociceptive Mechanisms to Reduce Intense Acute Swimming-Induced Muscle Pain in Mice. *PLoS One* 11**,** e0162267.

Borghi, S.M., Pinho-Ribeiro, F.A., Zarpelon, A.C., Cunha, T.M., Alves-Filho, J.C., Ferreira, S.H., Cunha, F.Q., Casagrande, R., and Jr, W.A. (2015). Interleukin-10 limits intense acute swimming-induced muscle mechanical hyperalgesia in mice. *Exp Physiol*.

Borghi, S.M., Zarpelon, A.C., Pinho-Ribeiro, F.A., Cardoso, R.D., Cunha, T.M., Alves-Filho, J.C., Ferreira, S.H., Cunha, F.Q., Casagrande, R., and Verri, W.A., Jr. (2014a). Targeting interleukin-1beta reduces intense acute swimming-induced muscle mechanical hyperalgesia in mice. *J Pharm Pharmacol* 66**,** 1009-1020.

Borghi, S.M., Zarpelon, A.C., Pinho-Ribeiro, F.A., Cardoso, R.D., Martins-Pinge, M.C., Tatakihara, R.I., Cunha, T.M., Ferreira, S.H., Cunha, F.Q., Casagrande, R., and Verri, W.A., Jr. (2014b). Role of TNF-alpha/TNFR1 in intense acute swimming-induced delayed onset muscle soreness in mice. *Physiol Behav* 128**,** 277-287.

Ruiz-Miyazawa, K.W., Pinho-Ribeiro, F.A., Borghi, S.M., Staurengo-Ferrari, L., Fattori, V., Amaral, F.A., Teixeira, M.M., Alves-Filho, J.C., Cunha, T.M., Cunha, F.Q., Casagrande, R., and Verri, W.A., Jr. (2018). Hesperidin Methylchalcone Suppresses Experimental Gout Arthritis in Mice by Inhibiting NF-kappaB Activation. *J Agric Food Chem* 66**,** 6269-6280.

Rustay, N.R., Wahlsten, D., and Crabbe, J.C. (2003). Assessment of genetic susceptibility to ethanol intoxication in mice. *Proc Natl Acad Sci U S A* 100**,** 2917-2922.
